# Investigating the Potential Effects of F-53B on Pulmonary Arterial Hypertension Through Network Toxicology, Molecular Docking, and In Vitro Validation

**DOI:** 10.3390/toxics14060477

**Published:** 2026-05-29

**Authors:** Lingling Xu, Yujie Ma, Zhenming Zheng, Fei Zou, Wenjun Li

**Affiliations:** Guangdong Provincial Key Laboratory of Tropical Disease Research, Department of Occupational Health and Occupational Medicine, School of Public Health, Southern Medical University, Guangzhou 510515, China

**Keywords:** F-53B, pulmonary arterial hypertension, network toxicology, molecular docking, chemokines

## Abstract

Pulmonary arterial hypertension (PAH) is a fatal vascular disorder with poor prognosis. 6:2 chloro-polyfluorooctane ether sulfonate (F-53B), a persistent environmental contaminant detected in humans, is known to be vasculotoxic, yet its association with PAH remains unexplored. This study aims to elucidate the mechanisms linking F-53B exposure to PAH by integrating network toxicology, molecular docking and in vitro experiments. Potential F-53B targets were predicted using ChEMBL, PharmMapper, and TargetNet. PAH-related genes were compiled from GeneCards, Online Mendelian Inheritance in Man (OMIM), Therapeutic Target Database (TTD), and GSE254617. We identified 42 key targets of F-53B-related PAH. Functional enrichment revealed terms such as inflammatory response and extracellular matrix. Protein–protein interaction (PPI) analysis identified five hub genes: CCL2, CXCL8, CCL5, CCR2, and CCL11. Molecular docking confirmed strong binding between F-53B and these core targets, with CCR2 showing the strongest affinity (−10 kcal/mol). Molecular dynamics simulations further verified stable binding to CCR2. In vitro experiments demonstrated that F-53B activated the CCL2/CCR2 axis and induced IL-1β, TNF-α, and IL-6 in HUVECs and RAW264.7 cells. This study reveals that F-53B is linked to PAH through dysregulation of chemokine signaling networks and induction of inflammatory cytokines. These findings suggest F-53B as a potential environmental risk factor for PAH and pinpoint potential targets for intervention.

## 1. Introduction

Pulmonary arterial hypertension (PAH) represents a challenging, incurable condition marked by pulmonary vascular remodeling, resulting in right-sided heart failure and early mortality [1]. Vascular remodeling is an irreversible histological lesion characterized by endothelial abnormalities, smooth muscle cell proliferation, abnormal fibroblast activation, inflammatory cell infiltration, and aberrant extracellular matrix modulation [2,3,4,5]. It has a prevalence of 15–50 cases per million, with an annual rate of roughly 5 cases for every million individuals [6,7]. Despite advances in therapeutic strategies, including drugs targeting the prostacyclin, endothelin-1, and nitric oxide pathways, the prognosis remains unfavorable, with a median survival of 5 to 7 years and a mortality rate approaching 57% [8]. Therefore, deciphering the triggers and mechanisms underlying PAH is essential for devising more effective therapies and improving patient outcomes.

Environmental exposure interacting with genetic susceptibility is recognized as an important contributor to PAH pathogenesis [9]. Perfluorooctane sulfonate (PFOS), a persistent per- and polyfluoroalkyl substance, has been widely restricted due to its pronounced bioaccumulation potential and adverse health effects [10]. In response, 6:2 chlorinated polyfluorinated ether sulfonate (Cl-PFESA; trade name F-53B) has been commonly utilized as an industrial alternative to PFOS, particularly for chromium mist suppression in Chinese electroplating operations [11]. Studies have confirmed the presence of F-53B in diverse environmental compartments (e.g., surface water, soil, and air) and human matrices (e.g., serum, urine, and breast milk) [12,13,14]. F-53B, with a 15.3-year half-life, appears in approximately 80% of human blood specimens and has been linked to various health risks, including impaired lipid homeostasis [15], dysregulated vascular function [16], endocrine disorders [17], and poor birth outcomes [18]. Moreover, toxicological studies have further demonstrated that F-53B can cause multi-organ toxicity. It induces neurotoxicity by inhibiting the V-ATPase-AMPK axis [19], disrupts liver lipid homeostasis by activating the PPAR pathway [20], and induces intestinal inflammation by disturbing gut microbiota [21]. Given its persistence and widespread detection in human blood, the specific risk of vascular toxicity warrants particular investigation. Emerging evidence indicates that F-53B induces vascular injury via endothelial dysfunction, inflammation, and smooth muscle cell ferroptosis and phenotype transition, thereby predisposing the aortic vasculature to remodeling [22,23]. In addition, F-53B exposure triggers endothelial-to-mesenchymal transition (EndMT) in the placental vasculature via the TGF-β/Snail signaling pathway, leading to placental vascular injury [24]. However, the role of F-53B exposure in the pathogenesis of PAH remains unknown. The established role of vascular remodeling as the central pathological feature of PAH, coupled with the evidence of F-53B-induced vascular remodeling in mice, provides a compelling rationale for hypothesizing a link between F-53B exposure and PAH pathogenesis.

Network toxicology, an interdisciplinary field integrating network pharmacology and systems biology, systematically deciphers the mechanisms of chemical toxicants by constructing “toxicant-gene-protein” networks [25]. It leverages multi-omics data to map toxicity pathways and efficiently identify the toxic targets of various chemicals. Complementarily, molecular docking predicts ligand-target interactions and binding affinity through free energy calculations. The convergence of network toxicology with molecular docking and molecular dynamics (MD) simulations offers a powerful approach for elucidating the mechanisms underlying toxicant-induced effects by identifying their potential molecular targets [26,27]. A recent study employing network toxicology and molecular docking identified elevated TNF-α and IL-6 as pivotal triggers in PFOS-induced PAH [28]. Building upon this mechanistic insight and the close physicochemical similarity between F-53B and PFOS [29], we hypothesize that F-53B might also elicit analogous toxic effects.

By leveraging a combined approach of network toxicology, molecular docking, MD simulations, and in vitro experiments, this study elucidates the mechanistic basis of F-53B-associated PAH and seeks to provide novel insights into its toxicological profile and inform future preventive strategies.

## 2. Materials and Methods

### 2.1. Study Design

**Figure 1:**
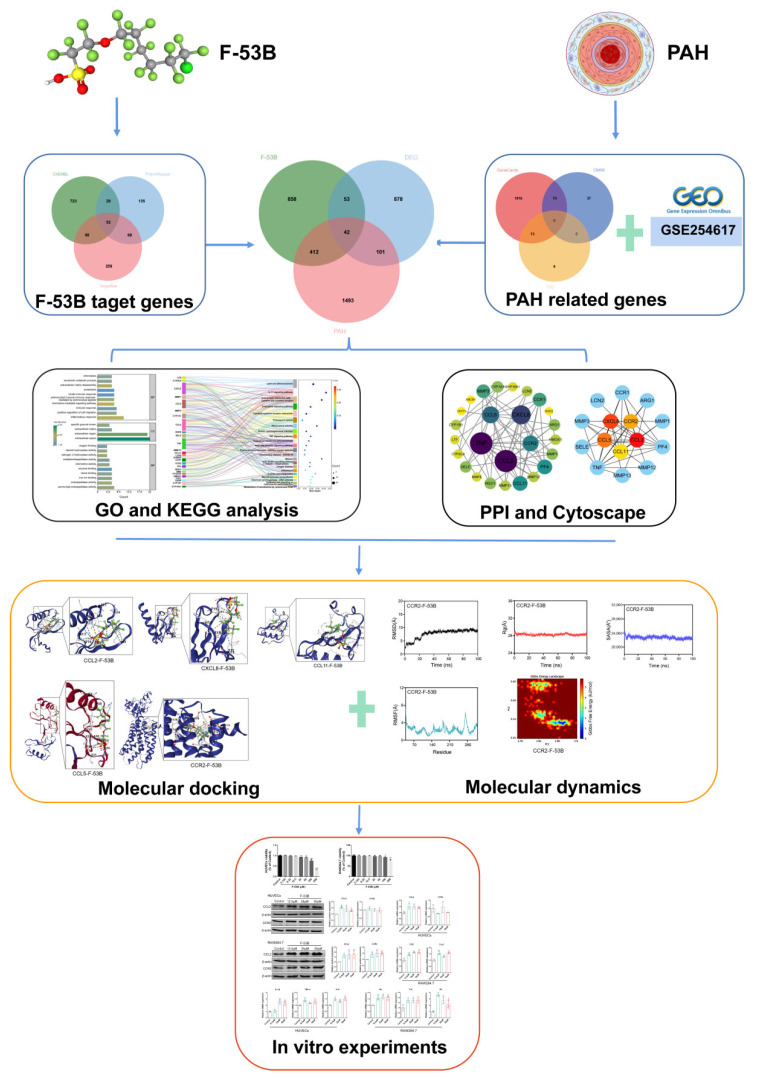
The workflow of this study.

First, potential targets of F-53B were retrieved from the ChEMBL, PharmMapper, and TargetNet databases. Simultaneously, genes associated with PAH were collected from GeneCards, Online Mendelian Inheritance in Man (OMIM), Therapeutic Target Database (TTD), and from the differentially expressed genes (DEGs) in the GSE254617 dataset. The intersecting genes from these sets, identified using a Venn diagram, were subsequently analyzed for Gene Ontology (GO) and Kyoto Encyclopedia of Genes and Genomes (KEGG) pathway enrichment using the DAVID database. A protein–protein interaction (PPI) network was then generated from these genes with the STRING database and analyzed in Cytoscape 3.10.2 to identify top hub genes using the CytoHubba plugin. Next, molecular docking was performed to validate the interactions between F-53B and the protein products of these hub genes. MD simulations were carried out to verify the stability of the F-53B-hub protein complexes. Finally, in vitro experiments were conducted to verify the predictions from the network toxicology analysis. The overall workflow is outlined in Figure 1.

### 2.2. Acquisition of Chemical Constituents and Target Prediction of F-53B

The simplified molecular input line entry system (SMILES) string and canonical 3D structural descriptors of F-53B were acquired from the PubChem database (https://pubchem.ncbi.nlm.nih.gov/, accessed on 11 September 2025). Target prediction of F-53B was performed using the ChEMBL (https://www.ebi.ac.uk/chembl/, accessed on 11 September 2025), PharmMapper (https://lilab-ecust.cn/pharmmapper/, accessed on 11 September 2025), and TargetNet databases (http://targetnet.scbdd.com/, accessed on 11 September 2025), with the SMILES string serving as the query term and the search restricted to Homo sapiens.

### 2.3. Identification of DEGs for PAH

DEGs were identified through transcriptomic data on PAH sourced from the Gene Expression Omnibus (https://www.ncbi.nlm.nih.gov/geo/, accessed on 15 September 2025). The dataset GSE254617 was selected using “Pulmonary arterial hypertension” as the search term. This dataset included explant lung tissue from 96 patients with PAH and 52 control individuals. The analysis of DEGs was carried out utilizing the “DESeq2” package in R software (version 4.3.1). Genes exhibiting an absolute log2 fold change (|log2FC|) ≥ 1, coupled with an adjusted *p*-value below 0.05, were classified as differentially expressed.

### 2.4. Identification of PAH-Associated Targets

A systematic search of the GeneCards (https://www.genecards.org/, accessed on 15 September 2025), OMIM (https://omim.org/, accessed on 15 September 2025), and TTD databases (https://db.idrblab.net/ttd/, accessed on 15 September 2025) was performed using “pulmonary arterial hypertension” as the key term to collect known disease-associated genes. These gene sets were then integrated with the DEGs from the PAH transcriptomic dataset (GSE254617) for subsequent analysis.

### 2.5. Enrichment Analysis

To characterize the biological functions of the overlapping targets, GO and KEGG pathway enrichment analyses were employed using the DAVID online bioinformatics resource (https://davidbioinformatics.nih.gov/, accessed on 25 September 2025), applying a significance level of *p* < 0.05. The GO analysis revealed the roles of targets across biological processes, cellular components, and molecular functions, and the KEGG analysis mapped these targets onto known signaling pathways. The results were integrated to infer underlying biological mechanisms.

### 2.6. PPI Network

The PPI network of the intersecting targets was generated using the STRING database (organism: Homo sapiens, interaction score ≥ 0.7). Cytoscape 3.10.2 was utilized for visualization of the network. The top central targets within this network were identified according to degree values using the cytoHubba plugin in Cytoscape 3.10.2.

### 2.7. Molecular Docking

The 3D conformation of F-53B underwent energy minimization via ChemOffice and was stored as a PDB file. The crystal structures of target proteins were retrieved from the RCSB PDB (http://www.rcsb.org/) using the following PDB IDs: CCL2 (3IFD), CXCL8 (5D14), CCL5 (5COY), CCR2 (5T1A), and CCL11 (7SCS). These were processed in PyMOL (https://pymol.org, accessed on 29 September 2025) to remove water molecules, phosphates, and heteroatoms. Molecular docking simulations were carried out using CB-Dock2 (https://cadd.labshare.cn/cb-dock2/, accessed on 29 September 2025) to assess receptor-ligand interactions. The conformation with the lowest Vina score was selected as the optimal pose, and its binding energy was calculated. Key interactions, including hydrogen bonds and hydrophobic contacts, were visualized in the 3D binding mode. For the validation of the established docking protocol, redocking experiments were performed using the native co-crystallized ligand of each target protein. The root mean square deviation (RMSD) value between the redocked pose and the original crystal conformation was calculated in PyMOL, which confirmed the reliability and feasibility of our molecular docking method.

### 2.8. Molecular Dynamics Simulation

MD simulations were performed using GROMACS 2022. The AMBER14SB force field was applied to the receptor proteins via the pdb2gmx tool. Ligand topology was generated with Sobtop 1.0 (dev3.1) using the GAFF2 force field, and atomic partial charges were assigned using the RESP method. Systems were solvated in a cubic TIP3P water box with a minimum solute–box distance of 1.0 nm. Na^+^ and Cl^−^ ions were added to a concentration of 0.15 M using the gmx genion tool to neutralize the systems and mimic physiological conditions. Long-range electrostatic interactions were treated with the particle mesh Ewald (PME) method using a cutoff of 1.0 nm, and bond lengths were constrained using the LINCS algorithm. Prior to MD simulations, energy minimization was performed in three stages (restrained minimization of water molecules, restrained minimization of counterions, and unrestrained minimization of the entire system), with a total of 3000 steps of steepest descent followed by 2000 steps of conjugate gradient minimization conducted across these stages. The systems were then equilibrated under NPT conditions at 310 K using a Nosé–Hoover thermostat and at 1 bar using a Parrinello–Rahman barostat. The simulations were conducted for 100 ns with a 2 fs time step under the NPT ensemble. During the simulations, the GROMACS tools gmx_rmsd, gmx_rmsf, gmx_gyrate, and gmx_sasa were used to calculate the RMSD, root mean square fluctuation (RMSF), radius of gyration (Rg), and solvent accessible surface area (SASA), respectively, in order to analyze system stability, structural changes, and solvent effects. Furthermore, the free energy landscape (FEL) was constructed using the gmx_sham tool based on the same RMSD and Rg metrics to characterize the thermodynamically favorable conformations.

### 2.9. Chemicals

F-53B [Cl(CF2)6O(CF2)2SO3K (CAS: 73606-19-6), purity ≥ 98%] was acquired from Hubei Qifei pharmaceutical&chemical Co., Ltd. (Hubei, China).

### 2.10. Cell Culture

Human umbilical vein endothelial cells (HUVECs) were obtained from the Chinese Academy of Sciences Cell Bank. The mouse macrophage cell line RAW264.7 was a generous gift from Professor Zhenlie Huang (School of Public Health, Southern Medical University, Guangzhou, China). The cell line was originally obtained from the American Type Culture Collection. Cells were maintained in DMEM (C11995500BT, Gibco, Waltham, MA, USA) containing 10% fetal bovine serum (FSP500, ExCell Bio, Shanghai, China) under standard culture conditions (37 °C, 5% CO_2_). For experimental treatments, cells were seeded at an appropriate density and allowed to adhere overnight. Subsequently, cells in the treatment groups were exposed to a range of F-53B concentrations, while the control group received vehicle (0.1% DMSO).

### 2.11. Cell Viability Assay for F-53B Exposure on HUVECs and RAW264.7 Cells

Based on previous study [22], HUVECs and RAW264.7 cells were exposed to various concentrations of F-53B (0, 3.125, 6.25, 12.5, 25, 50, 100, and 200 μM) for 24 h. Cell viability was determined with a Cell Counting Kit-8 (NCM Biotech, Suzhou, China) in accordance with the manufacturer’s guidelines. Briefly, each well received the CCK-8 reagent, which was then allowed to incubate for a predetermined period. Cell viability was quantified as a percentage of the vehicle-treated control group (0 μM F-53B).

### 2.12. Reverse Transcription Quantitative Polymerase Chain Reaction (RT-qPCR)

Upon 24 h of treating HUVECs and RAW264.7 cells with F-53B, the total RNA was harvested using TRIzol reagent (R401-01, Vazyme, Nanjing, China). Next, cDNA was synthesized through reverse transcription employing the Hifair^®^ III 1st Strand cDNA Synthesis SuperMix (11141ES60, Yeasen, Shanghai, China), utilizing exactly 1 μg of total RNA as starting material. Quantitative PCR was carried out on a Roche LightCycler^®^ 96 (Roche Life Science, Basel, Switzerland) employing Hieff^®^ qPCR SYBR Green Master Mix (No Rox) (11201ES08, Yeasen, Shanghai, China) adhering to the manufacturer’s protocols. The specific primers, commercially sourced from IGE Biotechnology, are detailed in Table S1. To determine gene expression levels, the 2^−ΔΔCt^ method was applied using ACTB/β-actin as the reference gene.

### 2.13. Western Blot

Protein extracts from HUVECs and RAW264.7 cells were prepared using RIPA buffer containing protease inhibitors (KGB5106-1, KeyGEN, Nanjing, China). Quantification of protein concentration was performed using a BCA assay kit (P0011, Beyotime, Shanghai, China). Equal amounts of protein were subjected to separation via 12% SDS-PAGE and subsequently electroblotted onto PVDF membranes (IPFL00010, Merck-Millipore, Billerica, MA, USA). Following blocking with 5% BSA, the membranes were probed with primary antibodies against MCP-1/CCL2 (#29547-1-AP, Proteintech, Wuhan, China), CCR2 (#R380703, Zenbio, Chengdu, China), and Beta Actin (#66009-1-Ig, Proteintech, Wuhan, China) overnight at 4 °C, followed by incubation with species-matched secondary antibodies. Protein bands were detected on a Li-COR Odyssey imaging system and their intensities were quantified with ImageJ software (version 1.8.0).

### 2.14. Statistical Analysis

Data are presented as mean ± standard deviation (SD) from experiments that were performed with three independent biological replicates (*n* = 3) and figures were created in GraphPad Prism 6.0. Differences between each treatment group and the control group were evaluated using one-way ANOVA followed by Dunnett’s post hoc test in SPSS software (version 22.0), with statistical significance set at *p* < 0.05.

## 3. Results

### 3.1. Identification of Target Genes in F-53B-Induced PAH

**Figure 2:**
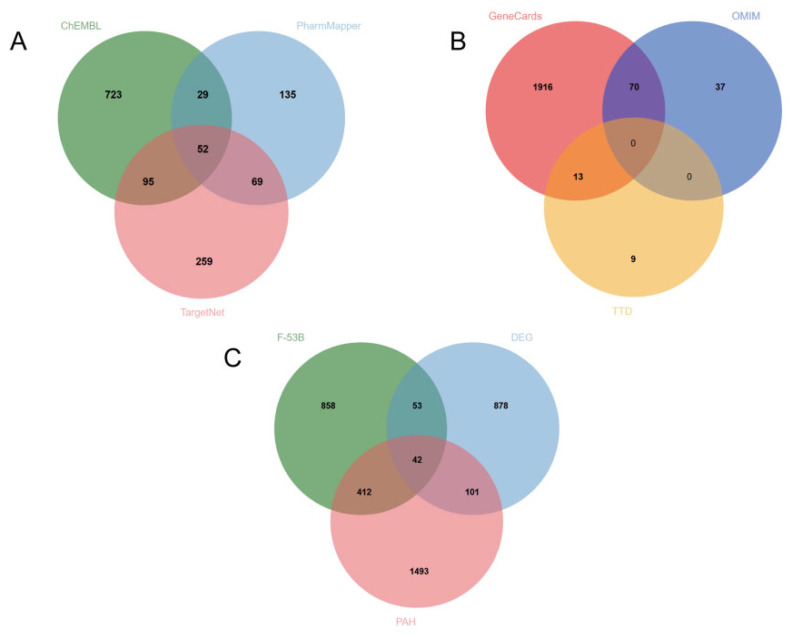
Identification of F-53B-associated disease targets in PAH. (**A**) Targets of F-53B were predicted using ChEMBL, PharmMapper, and TargetNet. (**B**) Venn diagram shows PAH-associated targets from Genecards, OMIM, and TTD. (**C**) Venn diagram shows the intersection of F-53B target genes, PAH-related genes, and DEGs, resulting in 42 shared genes.

Potential biological targets for F-53B were predicted through an in silico screen using ChEMBL, PharmMapper, and TargetNet, yielding 1362 candidates (Figure 2A). Concurrently, analysis of the GSE254617 dataset, which comprises lung transcriptomic data from 96 patients with pulmonary arterial hypertension and 52 controls, identified 1074 DEGs. In parallel, disease-associated genes were collected from GeneCards, OMIM, and TTD (Figure 2B). The intersection of these three gene sets using a Venn diagram revealed 42 common genes (Figure 2C), which represent potential core mediators linking F-53B exposure to PAH pathogenesis.

### 3.2. GO and KEGG Analysis of Targets

**Figure 3:**
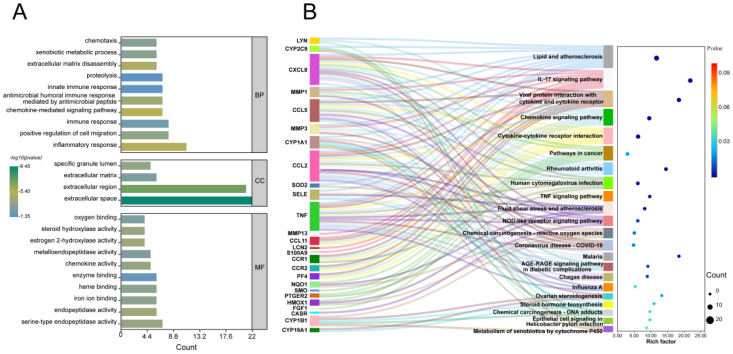
GO and KEGG enrichment analysis of the shared genes. (**A**) Histogram of GO terms enriched for the shared genes. (**B**) Sankey-bubble plot of KEGG enrichment analysis for the shared targets.

GO enrichment analysis of the 42 core genes revealed significant findings across three ontological categories (Figure 3A). Notably enriched biological processes included inflammatory and immune responses, as well as positive regulation of cell migration. Cellular components were particularly localized to the extracellular space, region, and matrix. Furthermore, key molecular functions such as serine-type endopeptidase activity, endopeptidase activity, and iron ion binding were enriched. KEGG pathway analysis identified significant signaling pathways closely associated with PAH, including the IL-17 signaling pathway, Chemokine signaling pathway, Cytokine–cytokine receptor interaction, and TNF signaling pathway, among others (Figure 3B). Collectively, these enrichment results suggest that F-53B-induced PAH may involve dysregulated immune-inflammatory responses and extracellular matrix changes.

### 3.3. Mapping the PPI Network and Identification of Core Targets

**Figure 4:**
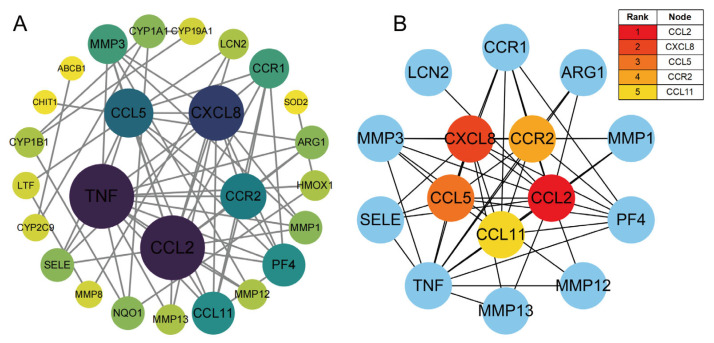
PPI network construction and identification of core PAH genes. (**A**) PPI network of the 42 overlapping genes. (**B**) Top hub genes ranked by node degree.

The 42 intersecting targets were subsequently submitted to the STRING database to obtain protein–protein interaction data, which were then imported into Cytoscape to construct a PPI network modeling their functional interplay (Figure 4A). The top five hub targets, ranked by node degree, were CCL2, CXCL8, CCL5, CCR2, and CCL11 (Figure 4B). These hub targets constitute a core functional module within the PPI network, suggesting their critical role in driving inflammatory pathways in PAH.

### 3.4. Molecular Docking of F-53B with Target Proteins

**Figure 5:**
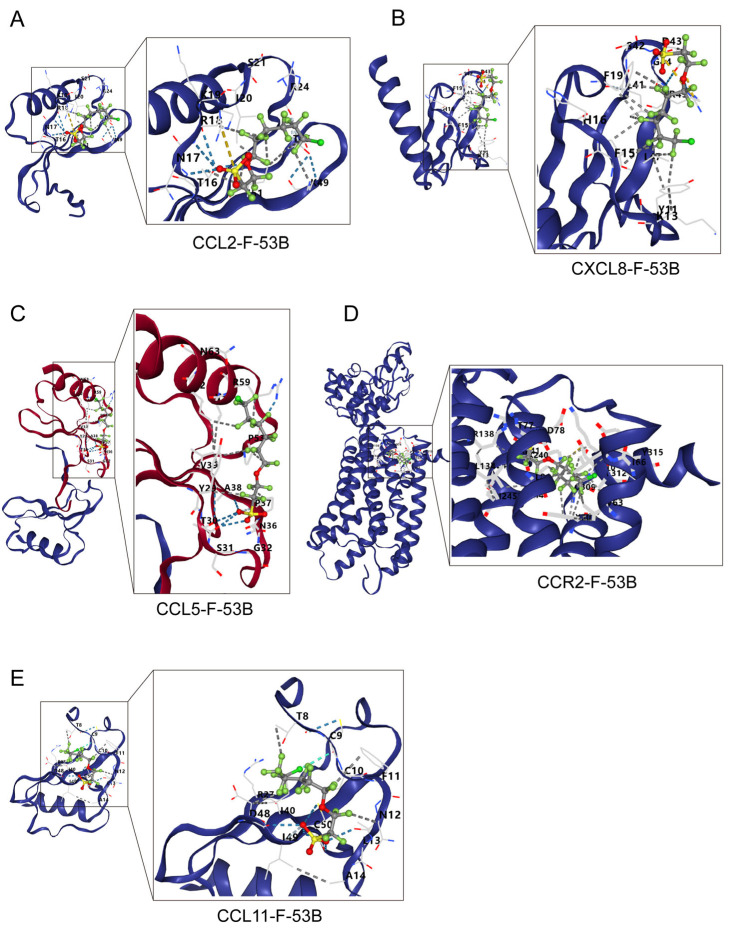
Molecular docking validation of F-53B binding to hub targets. (**A**) Docking results of CCL2 with F-53B. (**B**) Docking results of CXCL8 with F-53B. (**C**) Docking results of CCL5 with F-53B. (**D**) Docking results of CCR2 with F-53B. (**E**) Docking results of CCL11 with F-53B.

Molecular docking was carried out to validate the binding potential between F-53B and five key target proteins using their crystal structures: CCL2 (PDB: 3IFD), CXCL8 (PDB: 5D14), CCL5 (PDB: 5COY), CCR2 (PDB: 5T1A), and CCL11 (PDB: 7SCS). CB-Dock2 analysis yielded binding energies of −6.6, −6.6, −6.0, −10.0, and −5.9 kcal/mol for the complexes of F-53B with CCL2, CXCL8, CCL5, CCR2, and CCL11, respectively (Table 1). Lower energy values reflect stronger binding affinity. The results indicated robust interactions between F-53B and all five targets, with CCR2 showing the highest affinity (−10 kcal/mol). The binding poses were visualized using 3D representations generated by CB-Dock2 (Figure 5). After the docking analysis of F-53B, we conducted redocking verification. The calculated RMSD values were all within acceptable ranges, which demonstrated that our docking procedure was dependable. Detailed data are listed in Table S2. Collectively, these results offer structural evidence of direct F-53B binding to core PAH proteins.

### 3.5. Molecular Dynamics Simulation Analysis of the CCR2-F-53B Complex

**Figure 6:**
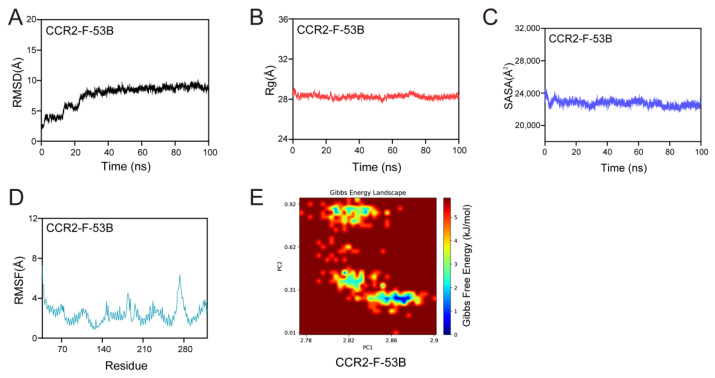
Molecular dynamics simulation of F-53B with CCR2. (**A**) Root mean square deviation (RMSD) values of the CCR2-F-53B complex. (**B**) Radius of gyration (Rg) values of the CCR2-F-53B complex. (**C**) Solvent accessible surface area (SASA) values of the CCR2-F-53B complex. (**D**) Root mean square fluctuation (RMSF) values of the CCR2-F-53B complex. (**E**) Free energy landscape (FEL) of the CCR2-F-53B complex. Blue regions represent lower free energy, and red regions represent higher free energy.

Given that CCR2 exhibited the strongest binding affinity to F-53B in molecular docking, this target was selected for subsequent 100 ns MD simulations to further verify its binding stability and dynamic interaction characteristics. RMSD is a critical metric for assessing the conformational stability of protein-ligand complexes and an indicator for measuring the degree of deviation between atomic positions and initial positions, with lower RMSD values indicating minimal structural deviation and enhanced stability. As shown in Figure 6A, the CCR2-F-53B complex reached equilibrium after 30 ns and subsequently maintained stable fluctuations around 9 Å, indicating stable binding of F-53B with the CCR2 target protein. Rg was employed to evaluate the compactness and overall structural integrity of the complexes. The Rg curve of the CCR2-F-53B complex remained stable at approximately 28 Å, reflecting a well-maintained compact structure (Figure 6B). SASA reflects the extent of protein surface exposure to the solvent. As illustrated in Figure 6C, the SASA of the CCR2-F-53B complex remained largely unchanged, further supporting stable binding between F-53B and CCR2. Additionally, RMSF was used to assess the flexibility of individual amino acid residues. As shown in Figure 6D, the CCR2-F-53B complex exhibited relatively low RMSF values, with most values below 4 Å, indicating high structural stability. FEL analysis was performed to identify the most energetically favorable conformations during the simulations. For the CCR2-F-53B complex, a single deep energy basin with sparse intermediate-energy conformations was observed, indicating a stable and rigid binding conformation (Figure 6E). Overall, these findings verify that F-53B binds stably to CCR2.

### 3.6. F-53B Activates CCL2/CCR2 Axis and Upregulates Inflammatory Cytokines

**Figure 7:**
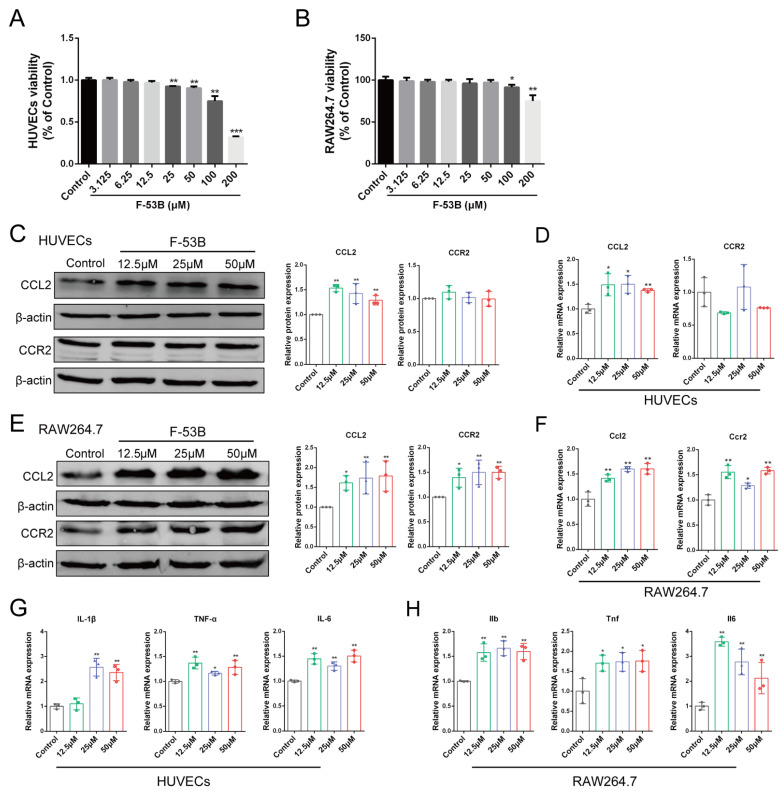
F-53B dysregulates the CCL2/CCR2 axis and induces inflammatory responses in HUVECs and RAW264.7 cells. (**A**,**B**) Assessment of the viability of HUVECs and RAW264.7 cells post-treatment with F-53B (0–200 µM for 24 h) via CCK-8 assay. (**C**,**D**) Western blot and RT-qPCR analyses of CCL2 and CCR2 expression in HUVECs following 24 h F-53B treatment. (**E**,**F**) Western blot and RT-qPCR analyses of CCL2 and CCR2 expression in RAW264.7 cells following 24 h F-53B treatment. (**G**) RT-qPCR analyses of IL-1β, TNF-α, and IL-6 expression in HUVECs after 24 h F-53B treatment. (**H**) RT-qPCR analyses of Il1b, Tnf, and Il6 expression in RAW264.7 cells after 24 h F-53B treatment. Data are from three independent experiments and presented as mean ± SD. * *p* < 0.05, ** *p* < 0.01, *** *p* < 0.001 compared to control group.

Notably, CCR2 functions as the specific receptor for CCL2 (one of the key hub targets identified earlier), so we next validated the regulatory effects of F-53B on both CCL2 and CCR2. Vascular endothelial dysfunction and inflammatory activation are central to the vascular remodeling in PAH. To model these processes, we used HUVECs to represent endothelial dysfunction and RAW264.7 cells to assess associated inflammatory responses. Initially, assessment of F-53B’s cellular toxicity was conducted via the CCK-8 assay. In HUVECs, cell viability was not significantly affected at concentrations up to 12.5 μM. However, it began to decrease significantly at 25 μM and dropped sharply at higher concentrations (Figure 7A). In RAW264.7 cells (Figure 7B), a marked decrease in viability was observed at 100 µM. Based on these cytotoxicity profiles, concentrations of 12.5, 25, and 50 μM were adopted for further study. Subsequently, the effects of F-53B on the expression of the key targets CCL2 and its receptor CCR2 were investigated. HUVECs exhibited increases in both CCL2 protein and mRNA levels following F-53B treatment, while CCR2 levels were unaltered compared with the control group (Figure 7C,D). RAW264.7 cells treated with F-53B exhibited increased protein and mRNA levels of both CCL2 and CCR2 compared with the control group (Figure 7E,F). Additionally, F-53B upregulated the IL-1β, TNF-α, and IL-6 mRNA levels in HUVECs (Figure 7G). A similar upregulation was observed in RAW264.7 cells (Figure 7H). Collectively, these results demonstrate that F-53B activates the CCL2/CCR2 axis and upregulates inflammatory gene expression, which may contribute to vascular inflammation and endothelial dysfunction in PAH.

## 4. Discussion

Preliminary studies have indicated potential toxicities in animals and humans and suggested that F-53B may possess greater bioaccumulative potential and hazard than PFOS [30,31]. Recent toxicokinetic studies in male mice estimated the oral bioavailability of 6:2 Cl-PFESA to be approximately 65%. Moreover, the low reference dose (RfD) of 0.02 ng/kg/day for 6:2 Cl-PFESA, together with over half of the assessed regions having a hazard quotient (HQ) > 1, indicates a considerable health risk [32]. In this study, we integrated multi-source databases to identify shared targets linking F-53B exposure to PAH. Target screening and PPI network analysis revealed that F-53B likely disrupts the “chemokine signaling-inflammatory response” axis through direct binding to core targets, including CCL2, CXCL8, CCL5, CCR2, and CCL11, thereby contributing to the development of PAH.

Our analysis identified 42 potential toxicological targets for F-53B-associated PAH. Functional enrichment analysis of these targets demonstrated that the association between F-53B and PAH may primarily involve dysregulated immune-inflammatory responses and the extracellular matrix. These biological processes are fundamental to pulmonary vascular remodeling [33,34]. Specifically, the significant enrichment of the inflammatory and immune response, along with positive regulation of cell migration, aligns with the pathological hallmark of PAH characterized by inflammatory cell infiltration and vascular smooth muscle cell proliferation [35]. The enrichment in extracellular matrix components and endopeptidase activity suggests F-53B may exert toxicity by modulating the vascular microenvironment, potentially through altered proteolytic processing and extracellular matrix disruption. This could facilitate abnormal vascular cell migration and matrix deposition, a critical process in forming the characteristic obstructive vascular lesions in PAH [36,37,38]. Consistent with this notion, experimental studies demonstrate that F-53B promotes vascular remodeling through the induction of vascular inflammation and extracellular matrix accumulation [22,23]. In addition, KEGG pathway analysis revealed that key signaling pathways, such as IL-17, Chemokine, and TNF signaling pathways, are well-established contributors to PAH pathogenesis. Studies have shown that serum IL-17 levels are significantly elevated in patients with systemic lupus erythematosus-associated PAH (SLE-PAH). IL-17 drives the pathogenesis of PAH by upregulating β-catenin [39] and inducing endothelial-mesenchymal transition [40]. Similarly, TNF-α was observed in both PAH patients and animal models. It acts by boosting superoxide levels, which reduces DNA methylation and enhances proliferation of pulmonary arterial smooth muscle cells (PASMCs) [41]. Additionally, inflammatory TNF-α signaling promotes smooth muscle cell proliferation by inhibiting bone morphogenetic protein receptor type II (BMPR-II), further aggravating vascular remodeling in PAH [42].

PPI network analysis identified five central hub genes (CCL2, CXCL8, CCL5, CCR2, and CCL11), underscoring the pivotal role of chemokine signaling in PAH pathogenesis. Moreover, molecular docking studies provided computational evidence supporting plausible direct interactions between F-53B and these hub proteins, with all complexes exhibiting substantial binding affinities (binding energies < −5 kcal/mol). Binding energy scores below −5.0 kcal/mol are interpreted as potential binding, and those below −7.0 kcal/mol are considered to denote strong binding [43]. MD simulation results further provided proof of stable binding between F-53B and CCR2, as reflected by RMSD, RMSF, Rg, and SASA values, along with energy basins in free energy landscape plots. Such stable molecular binding events may exert persistent effects on the functions of CCR2. Importantly, these MD simulation results lay a solid molecular foundation for subsequent in vitro functional validation experiments.

CCL2 and its primary receptor CCR2 are well-established mediators of monocyte/macrophage recruitment to sites of vascular injury in PAH. Suppression of CCL2/CCR2 signaling has been shown to inhibit vascular remodeling in experimental models [44]. A recent study demonstrated that CCL2 derived from resident interstitial macrophages recruits CCR2+ macrophages, which promote thrombospondin-1-mediated TGF-β activation, thereby driving vascular pathology in PAH [45]. Similarly, CCL5 drives vascular remodeling via the CCL5/CCR5 axis in macrophage-smooth muscle cell crosstalk [46] and also recruits T cells, initiating a self-perpetuating inflammatory cycle and exacerbating vascular injury [47]. In addition, CCL5 enhances hypoxia-induced contraction of intrapulmonary arteries through a GPR75-dependent mechanism in a mouse model of hypoxic pulmonary hypertension [48]. CXCL8 is known as interleukin-8 (IL-8). Evidence suggests that loss of BMPR-II function in pulmonary endothelial cells disrupts vascular integrity and upregulates proinflammatory mediators such as IL-8 [49]. This upregulation activates CXCR1/2 signaling, enhancing leukocyte recruitment and transmigration into the pulmonary vasculature [50]. CCL11 may drive PAH progression through the recruitment of eosinophils and their mediation of smooth muscle proliferation [51]. Collectively, the direct molecular interactions between F-53B and key chemokine-related proteins, coupled with the well-established roles of these hubs in driving vascular inflammation, immune cell recruitment, and remodeling, strongly implicate chemokine network dysregulation as a central mechanistic pathway linking F-53B exposure and PAH.

Given this central role of chemokine dysregulation, we focused on the biological validation of the CCL2/CCR2 axis, given its topological prominence and superior docking score with F-53B. Our in vitro results demonstrated that F-53B significantly upregulated CCL2 expression in both HUVECs and RAW264.7 cells. As a central driver of inflammation, CCL2 recruits monocytes, macrophages, and neutrophils, thereby amplifying and accelerating the inflammatory cascade [33,52]. Moreover, in murine PAH models, CCL2 derived from M2 macrophages has been identified as a key factor driving the proliferation of pulmonary artery smooth muscle cells, leading to vascular remodeling [53]. Furthermore, F-53B treatment enhanced CCR2 expression in macrophages, potentially priming them for hyper-responsiveness to CCL2 and creating a pathogenic feedback loop. This CCL2/CCR2 axis critically sustains immune cell activation and infiltration within the pulmonary vasculature, contributing to vascular remodeling [54]. Increased CCL2 expression favored M1 macrophage polarization, which increased the expression of inflammatory cytokines IL-1β, TNF-α, and IL-6 [55]. After exposure to F-53B, the upregulation of key inflammatory cytokines (IL-1β, TNF-α, IL-6) in both HUVECs and RAW264.7 cells establishes that F-53B induces a potent inflammatory response. This finding coherently supports the inflammatory pathway enrichments observed in our bioinformatics analysis. Prior studies have reported that F-53B activates the NLRP3 inflammasome and increases cytokines like IL-6 and IL-1β in the placenta and testis [56,57], underscoring its broad inflammatory potential. Serum IL-1β levels correlate with disease severity in patients [58]. In PAH models, macrophage-derived IL-1β, produced via caspase-8-dependent inflammasome activation, acts as a key mediator of perivascular inflammation [59]. Serum IL-6 levels are significantly elevated in PAH patients and negatively correlate with pulmonary function [60]. It contributes to disease progression by promoting the proliferation of PASMCs [61], and its inhibition has been shown to ameliorate PAH in experimental models [62]. Collectively, these findings suggest that F-53B may promote pulmonary vascular inflammation and remodeling through dysregulation of the CCL2/CCR2 chemokine axis and upregulation of inflammatory factors. This mechanism provides a potential link between F-53B exposure and the inflammatory pathogenesis of PAH.

Although this study broadens the perspective on F-53B as an environmental hazard, several limitations merit consideration. First, the findings are primarily computational and require rigorous experimental validation. Future studies that combine binding assays with functional investigations are necessary to confirm the direct interaction between F-53B and its targets, particularly the binding interaction with CCR2, as well as their physiological significance. The specific mechanisms by which F-53B exposure alters hub targets and induces inflammatory responses require further elucidation. Second, F-53B has accumulated extensively in environmental matrices with concentrations ranging from 1.11 ng/L to 112 μg/L in surface water, groundwater, and industrial wastewater [63,64,65]. Biomonitoring studies indicate that serum concentrations of F-53B in the general population range from 0.036 to 500 ng/mL (approximately 0.9 μM), and levels in occupationally exposed individuals can reach up to 5040 ng/mL (approximately 9 μM) [66]. The F-53B concentrations used in our in vitro experiments (12.5–50 μM) are higher than human serum F-53B levels. In addition, the use of a single in vitro system cannot fully reflect the multicellular complexity and chronic low-dose exposure characteristics of human environmental exposure. Therefore, future in vivo studies based on environmentally relevant concentrations and realistic human F-53B exposure levels are essential to determine the association between F-53B exposure and the development of PAH phenotypes, while also accounting for systemic responses. Third, prospective cohort studies are warranted to investigate the correlation between internal F-53B exposure levels and PAH incidence or severity in human populations.

## 5. Conclusions

In conclusion, our integrated analysis establishes a potential molecular connection between F-53B exposure and PAH pathogenesis, demonstrating a toxicological mechanism by which this environmental contaminant contributes to disease by disrupting chemokine networks, particularly the CCL2/CCR2 axis, and inducing inflammatory responses. These findings provide a preliminary framework for understanding the pulmonary vascular toxicity of F-53B, highlight candidate targets for future investigation, and underscore the need for strengthened pollution control.

## Figures and Tables

**Table 1:** Binding energies of ligands and receptors.

Ligand	Receptor	Binding Energy [kcal/mol]
F-53B	CCL2	−6.6
F-53B	CXCL8	−6.6
F-53B	CCL5	−6.0
F-53B	CCR2	−10.0
F-53B	CCL11	−5.9

## Data Availability

The raw data supporting the conclusions of this article will be made available by the authors on request.

## Supplementary Materials

The following supporting information can be downloaded at: https://www.mdpi.com/article/10.3390/toxics14060477/s1. Table S1: The primer sequences for qPCR; Table S2: RMSD value for redocking; Original Data S1: Raw image files for the Western blot.

## Abbreviations

The following abbreviations are used in this manuscript:

BMPR-II	Bone morphogenetic protein receptor type II
BP	Biological process
CC	Cellular component
CCK-8	Cell Counting Kit-8
CCL2	C-C motif chemokine ligand 2
CCL5	C-C motif chemokine ligand 5
CCL11	C-C motif chemokine ligand 11
CCR2	C-C chemokine receptor type 2
CXCL8	C-X-C motif chemokine ligand 8
DEGs	Differentially expressed genes
F-53B	6:2 chlorinated polyfluorinated ether sulfonate (trade name)
FEL	Free energy landscape
GEO	Gene Expression Omnibus
GO	Gene Ontology
HUVECs	Human umbilical vein endothelial cells
IL-1β	Interleukin-1 beta
IL-6	Interleukin-6
IL-17	Interleukin-17
KEGG	Kyoto Encyclopedia of Genes and Genomes
MD	Molecular dynamics
MF	Molecular function
NLRP3	NLR family pyrin domain containing 3
PAH	Pulmonary arterial hypertension
PPI	Protein–protein interaction
RMSD	Root Mean Square Deviation
RMSF	Root Mean Square Fluctuation
Rg	Radius of Gyration
RT-qPCR	Reverse transcription quantitative polymerase chain reaction
SASA	Solvent Accessible Surface Area
SMILES	Simplified molecular input line entry system
TNF-α	Tumor necrosis factor-alpha
