# Supplementary material for: Investigating the Potential Effects of F-53B on Pulmonary Arterial Hypertension Through Network Toxicology, Molecular Docking, and In Vitro Validation
Source: Toxics. 2026 May 29;14(6):477. doi: 10.3390/toxics14060477 (PMC13306741; doi:10.3390/toxics14060477)

Investigating the Potential Effects of F-53B on Pulmonary Arterial Hypertension Through Network Toxicology, Molecular Docking, and in vitro Validation

Lingling Xu, Yujie Ma, Zhenming Zheng,Fei Zou, and Wenjun Li

Guangdong Provincial Key Laboratory of Tropical Disease Research, Department of Occupational Health and Occupational Medicine, School of Public Health, Southern Medical University, Guangzhou 510515, China

Figure 7C

HUVECs

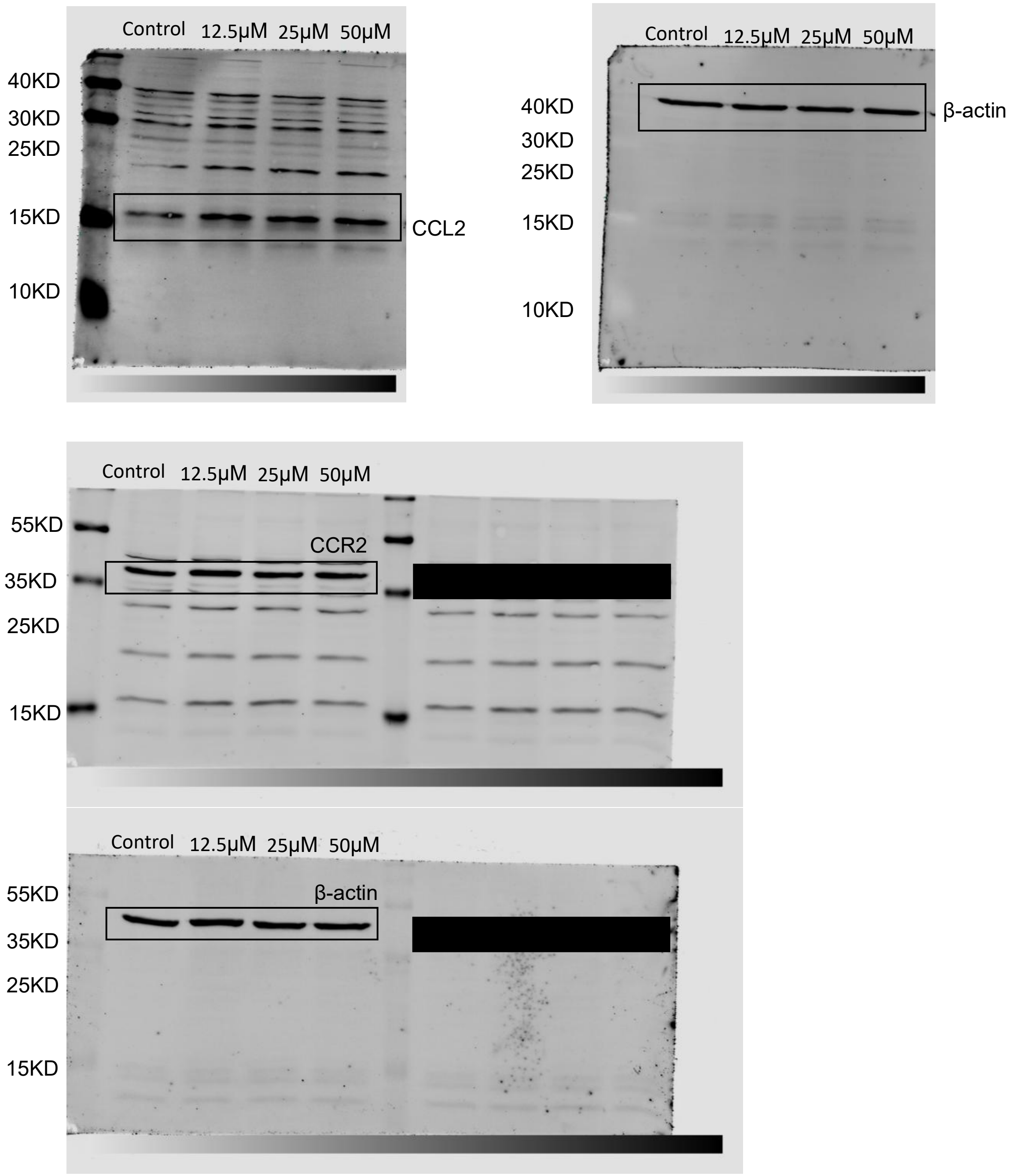

Figure 7E
RAW264.7

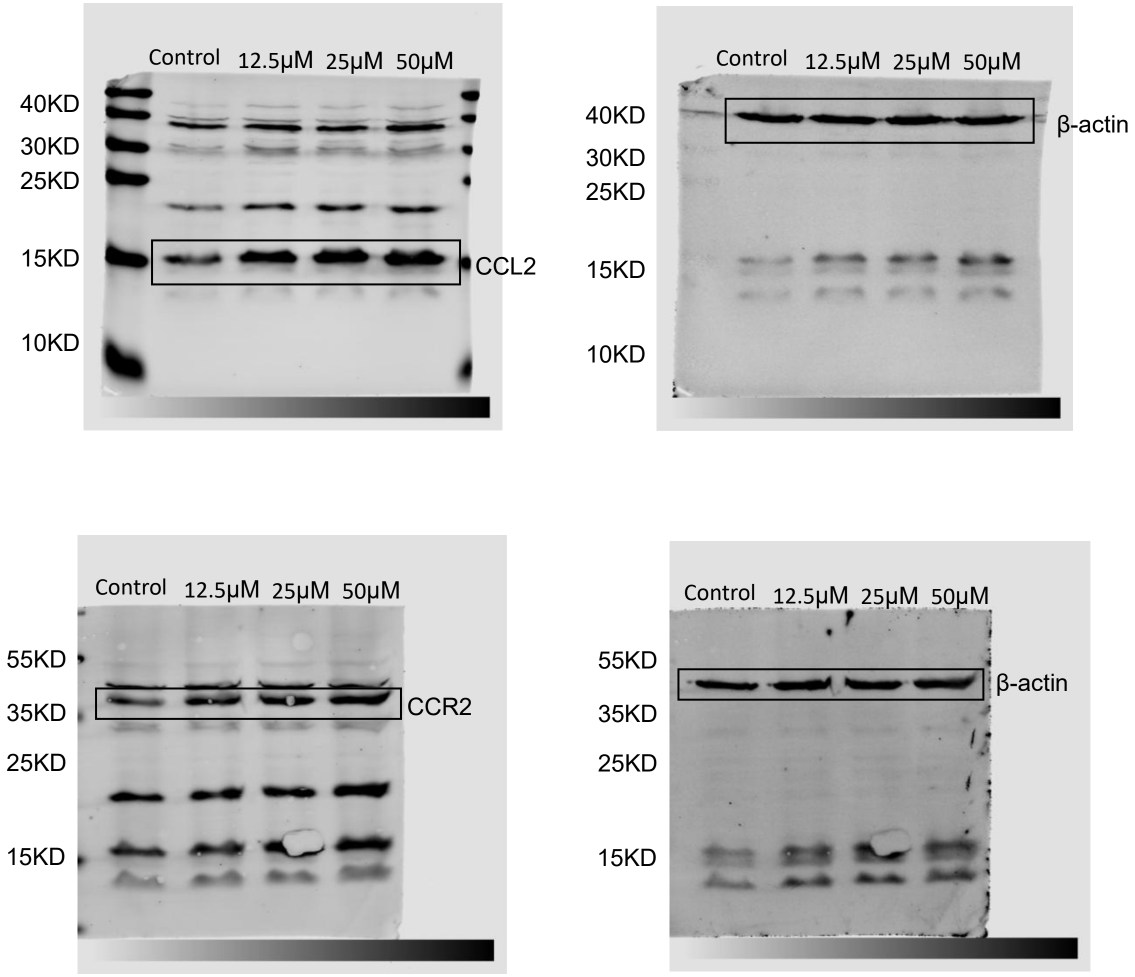

Supplement: Supplementary file 1 [file toxics-14-00477-s001.zip › toxics-4289299-supplementary/Original Data S1.pdf]
