# Supplementary material for: Investigating the Potential Effects of F-53B on Pulmonary Arterial Hypertension Through Network Toxicology, Molecular Docking, and In Vitro Validation
Source: Toxics. 2026 May 29;14(6):477. doi: 10.3390/toxics14060477 (PMC13306741; doi:10.3390/toxics14060477)
Supplement: Supplementary file 1 [file toxics-14-00477-s001.zip › toxics-4289299-supplementary/Supplementary Tables.pdf]

# Supplementary Table

Table S1 Primers for qPCR analysis.

| Gene name | Forward (5′-3′) | Reverse (5′-3′) |
|---|---|---|
| Human CCL2 | CAGCCAGATGCAATCAATGCC | TGGAATCCTGAACCCACTTCT |
| Human CCR2 | TACGGTGCTCCCTGTCATAAA | TAAGATGAGGACGACCAGCAT |
| Human IL1B | AGCTACGAATCTCCGACCAC | CGTTATCCCATGTGTCGAAGAA |
| Human TNF | CCTCTCTCTAATCAGCCCTCTG | GAGGACCTGGGAGTAGATGAG |
| Human IL6 | ACTCACCTCTTCAGAACGAATTG | CCATCTTTGGAAGGTTCAGGTTG |
| Human ACTB | CATGTACGTTGCTATCCAGGC | CTCCTTAATGTCACGCACGAT |
| Mouse Ccl2 | TTAAAAACCTGGATCGGAACCAA | GCATTAGCTTCAGATTTACGGGT |
| Mouse Ccr2 | ATCCACGGCATACTATCAACATC | CAAGGCTCACCATCATCGTAG |
| Mouse Ilb | GAAATGCCACCTTTTGACAGTG | TGGATGCTCTCATCAGGACAG |
| Mouse Tnf | GCAACTGTTCCTGAACTCAACT | ATCTTTTGGGGTCCGTCAACT |
| Mouse Il6 | CCACAGTCCTTCAGAGAGATACA | CCTTCTGTGACTCCAGCTTATC |
| Mouse β-actin | GTGACGTTGACATCCGTAAAGA | GCCGGACTCATCGTACTCC |

Table S2 RMSD value for redocking.

| Protein | PDB code | Redocking RMSD (Å) |
|---|---|---|
| CCL2 | 3IFD | 0.511Å |
| CCR2 | 5T1A | 0.354Å |
